# Characterization and Analysis of Clustered Regularly Interspaced Short Palindromic Repeats (CRISPRs) in Pandemic and Non-Pandemic *Vibrio parahaemolyticus* Isolates from Seafood Sources

**DOI:** 10.3390/microorganisms9061220

**Published:** 2021-06-04

**Authors:** Nawaporn Jingjit, Sutima Preeprem, Komwit Surachat, Pimonsri Mittraparp-arthorn

**Affiliations:** 1 Division of Biological Science, Faculty of Science, Prince of Songkla University, Hat Yai 90110, Songkla, Thailand; ma-pon@hotmail.com; 2 Microbiology Program, Faculty of Science Technology and Agriculture, Yala Rajabhat University, Muang District, Yala 95000, Yala, Thailand; sutima.p@yru.ac.th; 3 Division of Computational Science, Faculty of Science, Prince of Songkla University, Hat Yai 90110, Songkhla, Thailand; komwit.s@psu.ac.th; 4 Molecular Evolution and Computational Biology Research Unit, Faculty of Science, Prince of Songkla University, Hat Yai 90110, Songkhla, Thailand

**Keywords:** CRISPR, evolution, seafood, *V. parahaemolyticus*, hemolysin gene

## Abstract

*Vibrio parahaemolyticus* is one of the significant seafood-borne pathogens causing gastroenteritis in humans. Clustered regularly interspaced short palindromic repeats (CRISPR) are commonly detected in the genomes of *V. parahaemolyticus* and the polymorphism of CRISPR patterns has been applied as a genetic marker for tracking its evolution. In this work, a total of 15 pandemic and 36 non-pandemic *V. parahaemolyticus* isolates obtained from seafood between 2000 and 2012 were characterized based on hemolytic activity, antimicrobial susceptibility, and CRISPR elements. The results showed that 15/17 of the *V. parahaemolyticus* seafood isolates carrying the thermostable direct hemolysin gene (*tdh*+) were Kanagawa phenomenon (KP) positive. The Multiple Antibiotic Resistance (MAR) index ranged between 0.1 and 0.4, and 45% of the isolates have an MAR index ≥ 0.2. A total of 19 isolates were positive for CRISPR detection, including all *tdh*+ *trh*− isolates, two of *tdh*− *trh*+, and each of *tdh*+ *trh*+ and *tdh*− *trh*−. Four spacer types (Sp1 to Sp4) were identified, and CRISPR-positive isolates had at least one type of spacer homolog to the region of *Vibrio alginolyticus* megaplasmid. It is of interest that a specific CRISPR profile and spacer sequence type was observed with correlations to the hemolysin genotype (*tdh/trh*). Thus, these provide essential data on the exposure of foreign genetic elements and indicate shared ancestry within different genotypes of *V. parahaemolyticus* isolates.

## 1. Introduction

*Vibrio parahaemolyticus* is a Gram-negative halophilic bacterium which belongs to the family Vibrionaceae. It is an oxidase-positive, facultative anaerobic bacterium, similar to other members in the genus *Vibrio,* present in marine or estuarine environments [1]. Many *V. parahaemolyticus* strains are pathogenic and can cause gastroenteritis in humans due to consumption of raw or undercooked seafood [2]. It was discovered as a common cause of foodborne diseases in Japan in 1950 and is responsible for the world’s worst seafood-associated diarrhoea after new pandemic strains emerged in 1996 [3]. The virulence genes, *tdh* and *trh*, encoded for the thermostable direct hemolysin (TDH) and TDH-related hemolysin (TRH), respectively, are considered as virulence factors associated with *V. parahaemolyticus* hemolysis and cytotoxicity activity in the host, and have been used as pandemic group-specific markers together with group-specific *toxRS* PCR (GS-PCR) to characterize pandemic *V. parahaemolyticus* isolates associated with outbreaks [4]. In clinical *V. parahaemolyticus* isolates, most of them were pandemic strains that carry the *tdh* gene but not the *trh* gene. The *trh* gene shared approximately 67% sequence homology with the *tdh* gene [5]. There are five and two variants of the *tdh* gene (*tdh*1 to *tdh*5) and the *trh* gene (*trh*1 and *trh*2), respectively [6]. Several studies suggest that the *tdh* gene was acquired from other organisms by genetic transfer, involving plasmids and/or insertion sequence-like elements (named ISVs) [7]. The mechanisms of horizontal gene transfer are also suggested to possibly occur during the bacteriophage-mediated process. A whole-genome sequence comparison of pre-pandemic *V. parahaemolyticus*, obtained in 1985 with the genome of pandemic *V. parahaemolyticus* RIMD 2,210,633, isolated in 1996, confirmed that the evolutions of pandemic strains are driven by the acquisition of pathogenicity islands and foreign genetic elements [8]. A production of TDH is known to be correlated with the Kanagawa phenomenon (KP) and this could differentiate pathogenic from non-pathogenic *V. parahaemolyticus* isolates by growing bacteria on Wagatsuma blood agar [9]. This special agar contains NaCl at high concentrations, as well as fermentable carbohydrates, which promote the growth of *V. parahaemolyticus* and increase hemolysin production due to a decrease in pH.

The pathogenic potentials of *V. parahaemolyticus* were also linked with the resistance to antibacterial agents. The subtherapeutic or extensive use of antibiotics in aquaculture and human therapy is reported to be involved in the emergence of the multidrug resistant *Vibrio* spp., including *V. parahaemolyticus* [10]. Thus, the occurrence of antibiotic resistant *V. parahaemolyticus* in seafood should be evaluated to identify the risk potential involved in seafood consumption.

Clustered regularly interspaced short palindromic repeats (CRISPR) are detected in around half of the bacterial genomes, including *V. parahaemolyticus,* and is well known as a bacterial defence system against plasmids, bacteriophages, and foreign nucleic acids [11]. The CRISPR containing direct repeat (DRs) sequences with 24–47 bp nucleotideshave the symmetry to form a palindromic structure, and 21–72 bp of the spacer regions obtained from foreign genetic elements [12]. After the new spacers are sequentially added to the CRISPR loci, the polymorphism of repeat-spacer sequences occurs.

The number of spacers and nucleotide sequences are usually used for the strain typing of different bacterial isolates to organize and cluster them based on their spacer content similarity [13]. In our previous studies, an analysis of the CRISPR patterns combined with virulence genes (CRISPR-virulence typing) demonstrated epidemiological tracking in *Helicobacter pylori* [14]. In *V. parahaemolyticus*, CRISPR-virulence typing can be used to differentiate between *trh*1+ and *trh*2+ *V. parahaemolyticus* isolates from clinical sources [15]. A previous study demonstrated that the isolates that possessed the identical *trh* gene (*trh*1 or *trh*2) were classified in the same cluster. Although a combination of CRISPR spacer sequences with hemolysin genes (*tdh*, *trh*1, and *trh*2) was used for subtyping *V. parahaemolyticus*, the association between spacer groups and hemolysin genotypes has not been clearly addressed. Moreover, only a few studies document the presence of CRISPR among seafood isolates of *V. parahaemolyticus* [16,17].

Therefore, this study aimed to characterize *V. parahaemolyticus* isolates from seafood frequently consumed by people in Southern Thailand and analyze their CRISPR locus. The results obtained from this study may help us understand the characteristics and CRISPR locus in pandemic and non-pandemic *V. parahaemolyticus* seafood isolates.

## 2. Materials and Methods

### 2.1. Bacterial Isolates

A total of 51 seafood isolates and 4 clinical isolates (used for comparative purposes) of *V. parahaemolyticus,* obtained from Marine Microbiology Laboratory, Division of Biological Science, Prince of Songkla University, was utilized for this study (Table 1). All were confirmed as *V. parahaemolyticus* by specific-PCR, using *toxR* primers [18]. The isolates were kept in 20% glycerol stock and maintained frozen at −80 °C.

### 2.2. Detection of Hemolysin Genes

A single colony of *V. parahaemolyticus* was inoculated into tryptic soy broth (TSB) with 1% NaCl and incubated at 30 °C with shaking overnight. Genomic DNA was extracted using a boiling method, as described previously [19]. Briefly, the 1 mL of broth culture was centrifuged at 10,000× *g* for 5 min, washed twice, resuspended in 1 mL of sterile distilled water, and boiled for 10 min. The boiled culture was centrifuged at 20,000× *g* at 4 °C, 10-fold diluted, and used as templates to detect the *tdh* and *trh* genes. The virulence genes detection of *V. parahaemolyticus* performed by PCR targeted to the *tdh* gene using a forward primer (5′-GGTACTAAATGGCTGACATC-3′) and a reverse primer (5′- CCACTACCACTCTCATATGC -3′) to detect a 251 bp gene fragment and the *trh* gene using a forward primer (5′-GGCTCAAAATGGTTAAGCG-3′) and a reverse primer (5′-CATTTCCGCTCTCATATGC-3′) to detect a 250 bp gene fragment [20]. The PCR reaction was carried out with a reaction mixture consisting of 1.5 mM MgCl_2_, 0.2 mM dNTPs, 0.4 μM of each primer, 0.025 U of *GoTaq* DNA polymerase (Promega, Madison, WI, USA), and 2.0 μL DNA templates in a 20 μL volume. The PCR reactions were performed with a T100^TM^ Thermal Cycler (Bio-Rad, Hercules, CA, USA). The PCR process included initial denaturation at 96 °C for 5 min, followed by 35 cycles of denaturation at 94 °C for 1 min, annealing at 55 °C for 1 min, extension at 72 °C for 1 min, and a final extension at 72 °C for 7 min. Electrophoresis was performed on a 1% agarose gel and the PCR products were detected using a UV transilluminator.

### 2.3. Group-Specific PCR (GS-PCR)

To investigate pandemic isolates of *V. parahaemolyticus*, GS-PCR was carried out using a forward primer (5′-TAATGAGGTAGAAACA-3′) and a reverse primer (5′-ACGTAACGGGCCTACA-3′) to detect a 651 bp amplicon of the *toxRS* sequence of the new O3:K6 clone [3]. In each reaction of 20 μL, 0.2 μM of each primer, 0.125 mM dNTPs, 1.5 mM MgCl_2_, 4 μL of 5× reaction buffer, 0.025 U of *GoTaq* DNA polymerase (Promega, Madison, WI, USA), and 2.5 μL DNA templates were used. The amplification conditions consisted of an initial denaturation at 96 °C for 5 min, followed by 25 cycles of denaturation at 96 °C for 1 min, annealing at 45 °C for 2 min, extension at 72 °C for 3 min, and a final extension at 72 °C for 7 min. Electrophoresis was performed on a 1% agarose gel and the PCR products were detected using a UV transilluminator.

### 2.4. Determination of Hemolytic Activity

For a quantitative analysis of hemolytic activity, *V. parahaemolyticus* isolates were cultured in TSB with 1% NaCl and incubated at 30 °C with shaking overnight. After incubation, bacterial culture was transferred into a microcentrifuge tube and washed twice with phosphate buffer saline solution (PBS). Then, bacterial suspension was adjusted to 0.5 McFarland standard. A total of 190 μL of each strain were mixed with 10 μL of packed human red blood cells (HRBCs). Packed HRBCs were obtained after centrifugation at 4000 rpm for 10 min at 4 °C. After centrifugation, RBCs were washed 3 times with PBS and incubated at 37 °C for 5 h. Distilled water and PBS were added to prepared HRBCs as the positive and negative control, respectively. After incubation, the supernatant was obtained by centrifuge at 10,000 rpm for 10 min at 4 °C and the amount of hemoglobin released from the lysed HRBCs was measured at 540 nm [21]. The tests were performed in duplicate. The percentage of hemolysis was calculated using the following formula:

$$\%\;\mathrm{Hemolysis}=\frac{OD540\;of\;sample-OD540\;of\;negative\;control}{OD540\;of\;positive\;control-OD540\;of\;negative\;control}\times 100$$

### 2.5. Kanagawa Phenomenon (KP) Assay

For the Kanagawa phenomenon (KP) assay, bacteria were grown on Wagatsuma medium (BAM Media M178) containing 5% HRBCs. Plates were incubated at 37 °C for 35 h, and hemolytic zones around the colonies were observed. Isolates producing a clear hemolytic zone around the colonies were identified as Kanagawa phenomenon-positive, those with no hemolytic zone were identified as Kanagawa phenomenon-negative [22].

### 2.6. Antimicrobial Susceptibility Test

All isolates of *V. parahaemolyticus* were tested for susceptibility using a standard disk diffusion assay. Antibiotics will be used in this test according to Clinical and Laboratory Standards Institute (CLSI) guidelines [23], including ampicillin (10 µg), gentamicin (10 µg), erythromycin (15 µg), sulfonamide (300 µg), tetracycline (30 µg), ciprofloxacin (5 µg), sulfamethoxazole (25 µg), chloramphenicol (30 µg), norfloxacin (10 µg). The distribution of inhibition zone diameters was interpreted based on the Clinical and Laboratory Standards Institute (CLSI) guidelines. The Multiple Antibiotic Resistance (MAR) index was calculated using the formula MAR = a/b, where “a” is the number of antibiotics to which the test isolate was resistant, and “b” is the total number of antibiotics tested. A value greater than 0.2 indicates that the isolates were isolated from high-risk sources [24].

### 2.7. Determination of CRISPR Sequences

The presence of CRISPR element in *V. parahaemolyticus* was detected using the PCR method described previously [15]. Briefly, PCR was carried out using PCR mixture containing 1.5 mM MgCl_2_, 0.25 mM dNTPs, 0.25 μM of forward primer (5′-ATGCATTCCAAAGCTACCACTC-3′) and reverse primer (5′-GCCTACCAGATAGCAAGTGTCC-3′), 0.025 U of Takara *Ex Taq* DNA polymerase (Takara Biochemicals, Tokyo, Japan), and 2.0 μL of DNA templates in a 20 μL volume. The PCR process included initial denaturation at 94 °C for 1 min, followed by 30 cycles of denaturation at 94 °C for 1 min, annealing at 50 °C for 1 min, extension at 72 °C for 1 min, and a final extension at 72 °C for 5 min. The PCR products were purified using GenepHlow^TM^ Gel/PCR Kit (Geneaid, New Taipei, Taiwan) and sequenced (Macrogen, Seoul, Korea).

### 2.8. Analysis of CRISPR and CRISPR-Virulence Typing

CRISPR patterns, including the direct repeats (DRs) and spacers, were analyzed using the CRISPRfinder server available at https://crispr.i2bc.paris-saclay.fr/Server/ (accessed on 14 April 2020). The RNA secondary structure of repeats was predicted using RNA fold [25].

The homology analysis of spacer sequences was identified by submitting the spacer sequence to BLASTN (somewhat similar sequences). Additionally, CGView was used to visualize the positions of protospacer on foreign genetic elements [26]. ProgressiveMauve was used for multiple genome alignments to visualize conserved genomic regions [27].

CRISPR-positive isolates of *V. parahaemolyticus* were analyzed based on CRISPR sequences combined with virulence genes, *tdh* and *trh*. The dendrogram was constructed using BioNumerics 7.6 software (Applied Maths, Saint-Martens-Latem, Belgium) with the UPGMA algorithm, using the Dice similarity coefficient [15].

### 2.9. Statistical Analyses

The correlation analyses were analyzed by Pearson’s correlation test, using Statistical Package for Social Sciences (SPSS v. 17, IBM, Armonk, NY, USA). The correlation coefficient (r- value) was used to interpret the correlation and *p*-value ≤ 0.05 was considered statistically significant in comparative data.

## 3. Results

### 3.1. Detection of Virulence Genes and Hemolytic Activity

**Figure 1:**
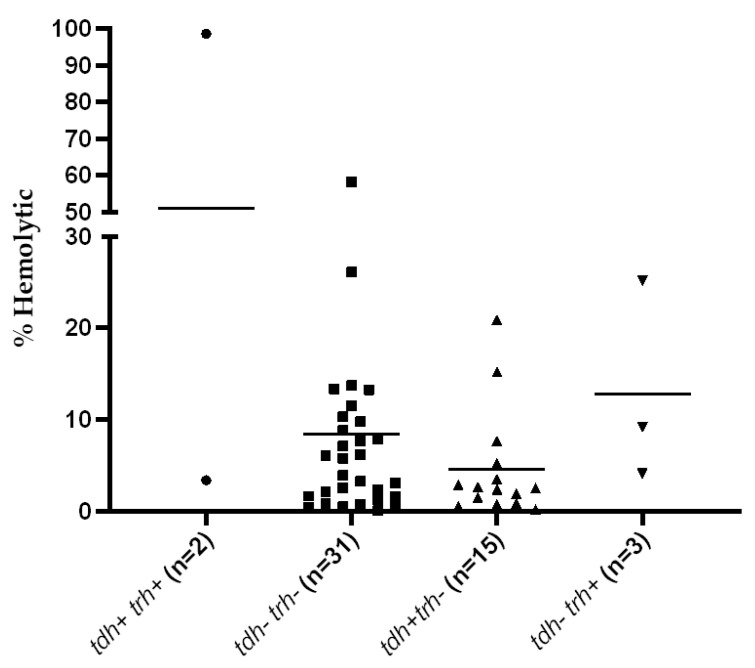
Quantitative analysis of the hemolytic activity of *V. parahaemolyticus* seafood isolates. Each point shows the mean value from duplicate experiments. Median values are shown by a horizontal line.

In this study, 51 isolates of *V. parahaemolyticus* isolated from seafood sources were grouped based on the presence of *tdh*, *trh*, and the pandemic marker genes detected by GS-PCR (Table 1). The percentages of hemolysis observed among *V. parahaemolyticus* isolates were varied and the correlation between hemolytic activity and the presence of the *tdh* gene was not found (r = 0.036, *p* = 0.802). The correlation between hemolytic activity and the presence of the *trh* gene was not done due to the small sample size. However, the greatest hemolytic activity (98%) was found in one of the *tdh*+ *trh*+ isolates (Figure 1). Furthermore, among 20 *tdh*+ isolates, 18 isolates exhibited β-hemolytic activity on Wagatsuma blood agar.

### 3.2. Characterization of Antimicrobial Susceptibility

All *V. parahaemolyticus* isolates from seafood were susceptible to chloramphenicol, whereas the highest resistance was recorded in response to ampicillin (100%) and followed by erythromycin (43.1%). It is of interest that 74.5% and 31.3% of the isolates demonstrated intermediate resistance to ciprofloxacin and norfloxacin, respectively (Table 2). An MAR index of 0.2 or above was shown by 23 isolates (45%) (Table 1). No significant correlation between the MAR index and the presence of the *tdh* gene (r = 0.096; *p* = 0.053), *trh* gene (r = 0.023; *p* = 0.871), or hemolytic activity (r = 0.112; *p* = 0.436) was observed.

### 3.3. Analysis of CRISPR Repeat Sequences and Patterns

A total of 19/51 (37.3%) of *V. parahaemolyticus* seafood isolates were positive for CRISPR (Table 1). Overall, the CRISPR element was significantly more prevalent in the *tdh*+ isolates and occurred less in the *tdh*− isolates (r = 0.832; *p* < 0.001). Two or three 28-bp direct repeats (DRs) were found and interspaced by spacers of 31 and/or 32 bp. (Table 3). 

**Figure 2:**
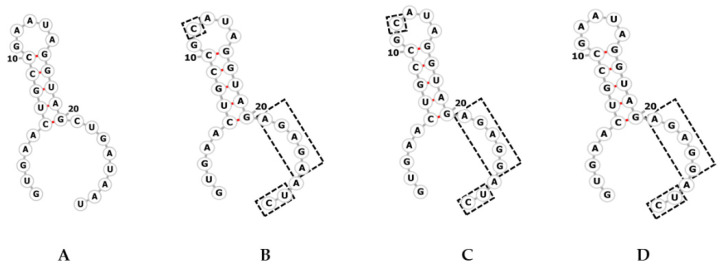
The secondary structures of four CRISPR repeats found in *V. parahaemolyticus* isolates. Predicted secondary structures were obtained using RNA fold. The dashed boxes indicate the sequence differences between typical repeat (**A**), and terminal repeat of *tdh*+ *trh*− (**B**), *tdh+ trh*+ and *tdh*+ *trh*− (**C**), and *tdh*− *trh*− (**D**) *V. parahaemolyticus* isolates, respectively.

Although the CRISPR repeat sequence is highly conserved, the polymorphisms within the terminal repeats were observed in this study (Table 3). The terminal repeats differed in a single nucleotide in the central region and 7 bases in the terminal region of predicted RNA secondary structures. However, these do not affect the based pairing of RNA structure (Figure 2).

### 3.4. Spacer Sequence Analysis

The spacer sequences analysis revealed 4 different CRISPR spacer types which were designated as Sp1 to Sp4 (Table 4). Sp1, Sp2, and Sp3 shared the best matched with 100, 96.7, and 87.1% homology to fragments of *V. alginolyticus* plasmid pL300 (Table 4). For Sp4, no homology to foreign genetic elements was detected.

It is of interest that the spacer type was found to be correlated with *tdh*/*trh* profile and at least one type of spacer homolog to the *V. alginolyticus* plasmid (Sp1 to Sp3) was shared by all CRISPR-positive *V. parahaemolyticus* isolates (Table 3). Further homology analysis of the spacer sequences against publicly available *V. parahaemolyticus* genomes showed the association of spacer type and *tdh*/*trh* profile, although virulence genes of some isolates were unknown (Table 5). These hits are located on a CRISPR array, as they were detected nearby cas gene(s), and some hits have also been addressed as CRISPR region.

### 3.5. Spacer Origin

**Figure 3:**
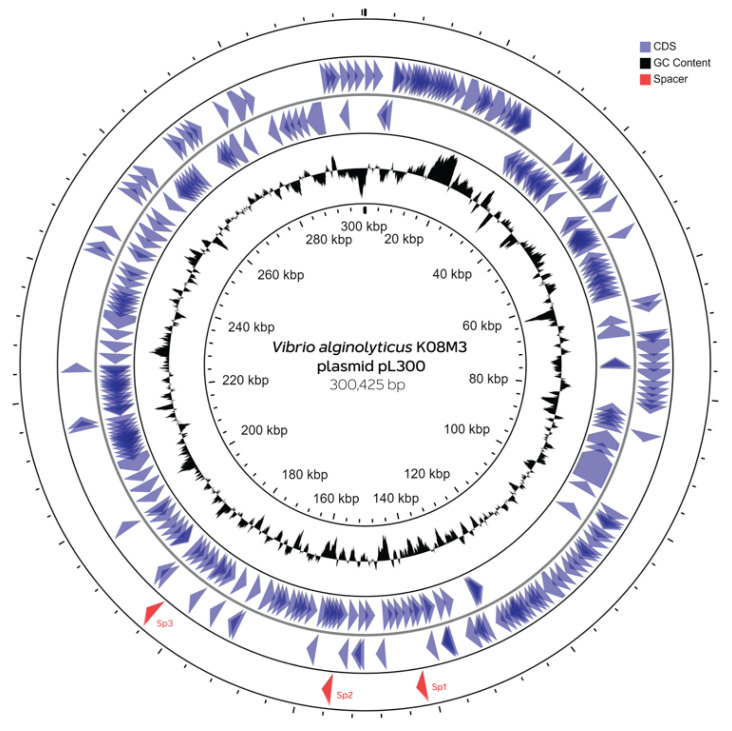
Distribution of protospacers along the representative plasmid pL300 of *V. alginolyticus* K08M3. The plasmid map is drawn from the GenBank entry CP017915.1 and ORFs are shown as arrows. Red arrows indicate the position of spacer-matching sequences (Sp1 to Sp3) found in the CRISPR region of *V. parahaemolyticus* analyzed in this study.

To further examine the finding of extrachromosomal origin of CRISPR spacers, the homology found among spacers was compared. This study found that Sp1, Sp2, and Sp3 sequences were matched with 6 mega-plasmid sequences of *V. alginolyticus* isolates, previously obtained from pipefish caught in the Kiel-Fjord, Germany, which ranged between 280 and 300 kbp in size [28]. Additionally, CRISPR spacers were mapped to represent Kiel *alginolyticus* megaplasmid, pL300, to locate the position of spacers. This study found that Sp1 to Sp3 spacers were distributed around pL300 (Figure 3). Sp1, Sp2, and Sp3 spacers were matched with the protospacer sequences of genes encoding for the modulator of FtsH protease Hfl, hypothetical protein, and DNA methyltransferase, respectively.

**Figure 4:**
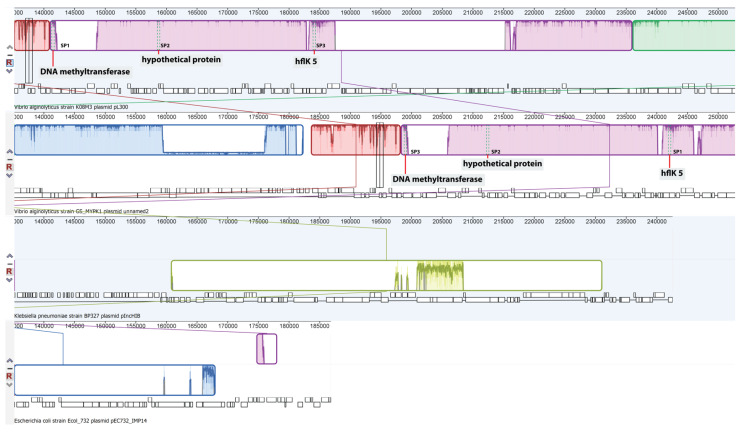
A zoomed-in view of the progressiveMauve alignment between *V. alginolyticus* pL300 megaplasmid (top) and other plasmids from the blast hit results. The protospacer regions are annotated and shown in the red vertical bar. Homologous genomes are indicated by the connected-with-lines collinear blocks.

The alignment between pL300 sequences and other plasmids was performed to identify plasmid identity. Apart from *V. alginolyticus* isolates from Kiel Fjord, this megaplasmid was highly homolog (98.6% identity; E-value of 0.0) to the MYPK1 plasmid of *V. alginolyticus* strain GS (accession number CP054703.1). Few similarities to other plasmid sequences of Enterobacteria, including *Escherichia coli*, *Klebsiella pneumoniae*, *Kluyvera ascorbata*, and *Citrobacter* sp. were observed (data not shown). The progressiveMauve alignment results showed the conservation regions among related ancestor plasmids (Figure S1). The conserved CDSs found among these plasmids included the essential genes required for plasmid conjugal transfer and integration (Table S1). A closer look at the protospacer regions among the four plasmids is shown in Figure 4.

### 3.6. CRISPR-Virulence Typing

**Figure 5:**
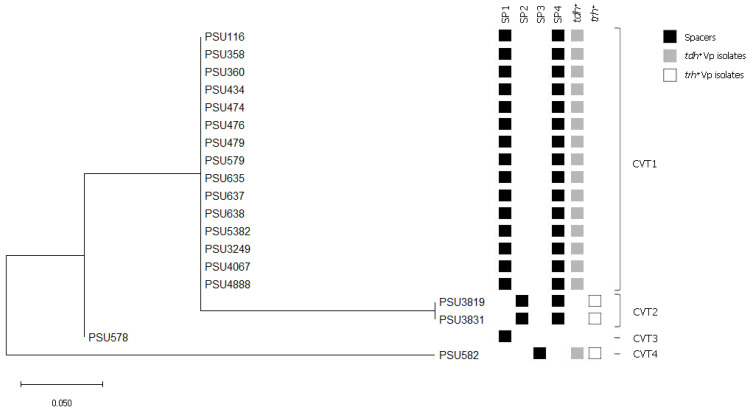
Dendrogram of the 19 CRISPR-positive *V. parahaemolyticus* seafood isolates. The maximum likelihood method was used for constructing the phylogenetic tree. The bar represents a 5% dissimilarity between the two sequences. Colors within the squares correspond to the presence of spacer and virulence genes.

In this study, a combination between CRISPR spacer sequence types and *tdh/trh* virulence genes of *V. parahaemolyticus* was used to create a dendrogram. CRISPR-virulence typing profiles of *V. parahaemolyticus* were organized into 4 CRISPR-virulence types (CVTs) (Figure 5). The isolates within the same CVT cluster composed identical virulence profiles and spacer content. 

The pandemic isolates with *tdh*+ *trh*− genotype was classified in the CVT1 cluster, which contained spacer type 1 and 4, and this was identical to that observed in the reference strain of *V. parahaemolyticus* RIMD 2210633. The *tdh*− *trh*+ and *tdh*− *trh*− isolates were classified in the CTV2 and CTV3 clusters, respectively. The differences between CVT1, CVT2, and CVT3 clusters were due to deletion or addition of a single spacer. It is of interest that the *tdh*+ *trh*+ isolate in the CVT4 cluster did not have any close association with others. Nevertheless, all 4 clusters shared spacers matched with pL300 of *V. alginolyticus,* suggesting that these isolates encounter this plasmid before they are diverged.

## 4. Discussion

In this study, 51 seafood isolates of *V. parahaemolyticus* previously, obtained from various cities in Southern Thailand between 2000 and 2012, were characterized for their hemolytic characteristics, antibiotic resistance, and CRISPR. All experiments included four hemolytic genotypes, pandemic and non-pandemic, of *V. parahaemolyticus* isolates. Although environmental and seafood isolates rarely contained *tdh*/*trh*, the presence of these isolates has been previously reported [29]. 

TDH- or TRH-producing *V. parahaemolyticus* are strongly associated with gastroenteritis. In this study, the result of a quantitative hemolysis assay against human blood demonstrated various levels of hemolytic activity exhibited by seafood isolates, and the highest hemolytic activity was detected in a *tdh*+ *trh*+ isolate. A previous study reported that temperature and culture density were the significant factors that affect the hemolysin activation of *V. parahaemolyticus* [30]. The gastroenteritis also showed a strong correlation with the KP, which is a type of β-hemolysis on the special blood agar and is induced by TDH [31]. A previous study reported that almost all environmental *V. parahaemolyticus* are *tdh*-negative and thus negative for KP [32]. In this work, two of the KP-negative *V. parahaemolyticus* isolates were positive for *tdh* gene. The previous study suggested that the isolates may possess a single copy of the *tdh* gene and therefore hemolysin was expressed at a low level [33]. Moreover, the hemolytic activity of *V. parahaemolyticus* may be phenotypically varied due to the sequence variation of hemolysin genes, which affects their promoter activities [6,34].

The increase in antibiotic-resistant bacteria is a major concern worldwide. Aquatic ecosystems could easily facilitate the exchange of mobile genetic elements among *V. parahaemolyticus* [35]. All *V. parahaemolyticus* isolates were resistant to ampicillin which is not recommended empirically to treat *V. parahaemolyticus* infection [36]. The ampicillin resistance found in this study correlates with other previous reports and this is not a new incident for ampicillin resistance among *Vibrio* spp. [37]. All *V. parahaemolyticus* isolates in this study were susceptible to chloramphenicol, which was the first truly broad-spectrum, and a high susceptibility to tetracycline (96.3%), sulfonamide (96.3%), and trimethoprim/sulfamethoxazole (67.2%) was observed. Interestingly, 72.7% and 34.5% of the isolates showed intermediate resistance to ciprofloxacin and norfloxacin, respectively which are the antimicrobial agents recommended to treat human infections [38]. This result was consistent with other studies [39]. The high level of intermediate resistance to both antibiotics may also tend to become resistant. Additionally, four isolates (PSU635, PSU3858, PSU4446) were identified as multidrug resistant (MDR) as they resist at least one agent in three or more antimicrobial classes [40]. This could lead to untreatable illnesses and human health risks. The MAR index value observed in this study indicated the differences in the original sources of the isolates. In this study, nearly half of the *V. parahaemolyticus* isolates had an MAR index ≥ 0.2, which indicated that these isolates originated from high-risk sources that have been exposed to antibiotics, such as humans and aquaculture, and seafood might be considered as a possible vehicle for pathogen transmission. Previous studies have reported that the *V. parahaemolyticus* isolates from seafood showed an MAR index > 0.2 [41,42]. Thus, antimicrobial resistance should continue to be monitored to ensure seafood safety. 

CRISPR-Cas system is known as a bacterial adaptive immune system against conjugative plasmids, or phages. The virulence and genotypes of *V. parahaemolyticus* are reported to be associated with CRISPR/Cas evolution [17]. This study found that all pandemic *V. parahaemolyticus* isolates were identified as CRISPR positive, suggesting the association between CRISPR and the pathogenic evolution of pathogenic *V. parahaemolyticus*. Previous reports demonstrated high percentages of *tdh+* (97.4%) and *trh*+ (47%) *V. parahaemolyticus* positive for CRISPR detection, with few reports demonstrating an association with the *tdh*− or *trh*− genotypes [15,17]. The result of this study corroborates earlier findings; however, we could detect CRISPR in two of the *tdh*− *trh*^+^ and one of the *tdh*− *trh*− isolates. The presence of CRISPR in the *tdh*− isolates were also reported in a previous study [41]. The CRISPR loci detected in this study is located on the *V. parahaemolyticus* island-7 (VPAI-7) region of chromosome 2, which encodes the *tdh* gene and are closely associated with *tdh*+ *V. parahaemolyticus* [43,44,45], thus this may be the reason why this CRISPR loci is more prevalent in the *tdh*+ isolates. Additionally, no significant correlation between the presence of CRISPR and hemolytic activity, or antimicrobial resistance, was observed (*p* > 0.05).

In this study, the DRs detected in CRISPR loci were 28-bp in length and its sequences have also been reported [16]. These characteristics were identical to those found in the reference strain of *V. parahaemolyticus* RIMD 2210633. In 2019, Baliga et al. analyzed the DRs of 200 *V. parahaemolyticus* genomes available in the database and found that 92% possessed a single type of repeat (RU-1) and the minimal set of Cas proteins [16]. The presence of similar DRs was observed in our recent study. However, the CRISPR primers used in this study did not target the Cas genes. Thus, the minimalistic type of CRISPR-Cas system could not be addressed here. Although a previous study reported 3–5 repeat units in the CRISPR loci of *V. parahaemolyticus*, we demonstrated 2 repeat units in the *tdh*+ *trh*+ and the *tdh*− *trh*− isolates. The presence of 2 DRs have been reported among *tdh*+ *trh*+ isolates from clinical samples [15,17]. Most isolates available in the public databases possess *tdh*+ *trh*− genotype, which may acquire more spacers than others. Moreover, further studies are necessary to clarify the types of CRISPR-Cas systems and their activities in the defence system against foreign mobile genetic elements.

This study found that the sequences of CRISPR repeats found among *V. parahaemolyticus* isolates were not highly identical, especially at the terminal position, however, this does not affect the RNA secondary structures. The polymorphisms in terminal sequences among the isolates in different genotypes were also observed. The variations within the central and terminal repeat sequence were also observed in the CRISPR loci of *Streptococcus thermophilus,* which suggested that the sequence degeneracy at the 3′-end of the terminal repeat is probably due to the homologous recombination events of DRs [46]. A degenerate repeat is required for the polarized spacer acquisition process [47].

BLAST comparisons of spacers identified matches between spacers and foreign plasmid sequences, which demonstrated the horizontal transfer of *V. alginolyticus* plasmid to *V. parahaemolyticus*. Most of the spacer sequences found in this study were similar to those reported in other *V. parahaemolyticus* isolates from seafood sources [16]. We have previously discussed the similarity of CRISPR spacers found in clinical isolates of *V. parahaemolyticus* with the *V. alginolyticus* plasmids [15]. In 2020, all of these plasmids were published as the megaplasmids associated with the Kiel isolates of *V. alginolyticus* [28]. In continuation of our previous discussion, the spacer sequence and origin were characterized and analyzed in a recent study.

In this study, 3 CRISPR spacers (Sp1 to Sp3) match the coding sequences of *V. alginolyticus* megaplasmid pL300, and one of these spacers (Sp4) was unique, as it demonstrated no homology to any sequences in the databases. It is often difficult to find sequence homology to spacer sequences because of the limited number of sequence information databases of plasmids and prokaryotic viruses [48]. All *V. parahaemolyticus* isolates included at least one spacer perfectly matching some region of pL300. One of the homology searches revealed the perfect match between CRISPR spacer and plasmid region, encoded for DNA methyltransferase, an enzyme required to protect DNA from restriction digestion [49]. This gene has been previously reported to be specifically targeted by CRISPR array against the virus to prevent successful host infection and lysis [50]. It is therefore conceivable that *V. parahaemolyticus* has adapted to its own evolution by avoiding the integration of foreign DNA from *V. alginolyticus*. Moreover, the results obtained by Progressive Mauve analysis demonstrated that pL300 may originate via the insertion of multiple regions of foreign DNA from bacteria in family Enterobacteriaceae. These conserved regions were found to encode essential genes of conjugative plasmids [49]. In addition, the evolution of conjugative plasmids could be driven through the CRISPR preventive mechanism.

CRISPR spacers can protect against foreign plasmid integration and phage invasions. The influence of these mobile genetic elements on the phenotypes and genotypes of marine *Vibrio* have been reported. Previous studies demonstrated a strong association of filamentous phages, f237, with recent pandemic strains of *V. parahaemolyticus*. This phage possesses a unique ORF8 open reading frame that is found only in recent pandemic isolates, and the Zot (Zonula occludens toxin)-like toxin previously described in the *V. cholerae* is reported to be encoded in the ORF7 [51,52]. A match was found between the CRISPR spacer region of *V. anguillarum* PF7 with a *zot-*encoding prophage in *V. anguillarum* PF4, indicating the contribution of CRISPR to the phage resistance of this bacterium [53]. In this study, no homologous between the CRISPR spacer and any phage regions or endogenous genomic sequences located outside the CRISPR array were found.

The results of this study indicated the association between spacer groups and hemolysin genotypes. Thus, a combination between CRISPR spacer sequences and the presence of hemolysin genes was used to cluster the *V. pahaemolyticus* isolates. As expected, a close relationship between the *tdh*+ *trh*− and the *tdh*− *trh*+ isolates were found in this study. The *tdh*− *trh*+ isolates were reported to be involved significantly in the pre-pandemic outbreaks, whereas the *tdh*+ *trh*− isolates are involved in both pre-pandemic and pandemics [8]. The *tdh*− *trh*− isolate was presented as the possible origin of the *tdh*+ *trh*− *V. parahaemolyticus* and the *tdh*+ *trh*+ isolates were phylogenetically distant to others. Since fewer spacers are less efficient in the protection against foreign genetic elements, further evolutional study is needed to explain this situation. In addition, the position of a spacer in a CRISPR loci indicates the historical timeline of *V. parahaemolyticus* exposure to foreign genetic elements [54].

## 5. Conclusions

An analysis of hemolysin genotypes in association with CRISPR loci can help to reveal the genome evolution of *V. parahaemolyticus*. Our characterization and in-depth analysis of CRISPR among seafood isolates of *V. parahaemolyticus* showed that these isolates exhibit various virulence phenotypes, and some isolates have become resistant to many antibiotics. More importantly, similar types of CRISPR repeats and spacer sequences were found in isolates harboring the same hemolysin genotypes (*tdh* and/or *trh*) and all CRISPR-positive isolates shared spacer(s) homolog to the similar plasmid of *V. alginolyticus*. In addition, the position of a spacer in a CRISPR loci could indicate an ancestry interaction of *V. parahaemolyticus* with a foreign genetic element. An in-depth analysis of CRISPR-Cas loci, combined with virulence genes, could provide important data for the evolutionary study of *V. parahaemolyticus*.

## Figures and Tables

**Table 1:** *V. parahaemolyticus* isolates used in this study and their characteristics.

Hemolysin Genotype (*n*)	Isolates	Year of Isolation	Source	GS-PCR *	Kanagawa Phenomenon	MAR * Index	CRISPR-PCR
Seafood isolates							
*tdh*+ *trh*− (15)	PSU166	2000	Hard clam	+	+	0.2	+
	PSU358	2001	Mussel Mussel Mussel Mussel Hard clam	+	+	0.2	+
	PSU360			+	+	0.2	+
	PSU434			+	+	0.2	+
	PSU474			+	+	0.2	+
	PSU476			+	+	0.1	+
	PSU479	2002	Hard clam	+	+	0.1	+
	PSU579		Cockle	+	+	0.2	+
	PSU635		Mussel	+	+	0.3	+
	PSU637		Mussel	+	+	0.2	+
	PSU638		Mussel	+	+	0.2	+
	PSU3249	2006	Mussel	+	−	0.1	+
	PSU4067	2008	Cockle	+	+	0.1	+
	PSU4888	2010	Hard clam	+	+	0.1	+
	PSU5382	2012	Shellfish	+	+	0.1	+
*tdh*− *trh*+ (3)	PSU3819 PSU3831	2007	Crab Fish	−	−	0.2	+
				−	−	0.2	+
	PSU5124	2011	Shrimp	−	−	0.1	−
*tdh*+ *trh*+ (2)	PSU582	2002	Cockle	−	+	0.2	+
	PSU4413	2008	Cockle	−	−	0.1	−
*tdh*− *trh*− (31)	PSU513 PSU571 PSU576 PSU578	2002	Cockle Hard clam Cockle Cockle	−	−	0.1 0.1	−
				−	−	0.1	−
				−	−	0.1	+
	PSU811	2003	Mussel	−	−	0.1	−
	PSU2463 PSU2467 PSU2471	2005	Cockle Cockle Cockle	−	−	0.2	−
				−	−	0.2	−
				−	−	0.2	−
	PSU3103 PSU3200 PSU3362 PSU3365	2006	Hard clam Cockle Mussel Cockle	−	−	0.1 0.2 0.2 0.1	−
	PSU3858 PSU4055 PSU4058 PSU4062 PSU4075 PSU4091 PSU4094	2008	Octopus Mussel Hard clam Cockle Hard clam Cockle Cockle	−	−	0.4	−
				−	−	0.1	−
				−	−	0.1	−
				−	−	0.2	−
				−	−	0.1	−
				−	−	0.1	−
				−	−	0.1	−
	PSU4415 PSU4418 PSU4425 PSU4446 PSU4459 PSU4460 PSU4575	2009	Cockle Cockle Cockle Cockle Cockle Cockle Cockle	−	−	0.1	−
				−	−	0.1	−
				−	−	0.1	−
				−	−	0.4	−
				−	−	0.1	−
				−	−	0.1	−
				−	−	0.1	−
	PSU4869 PSU4879 PSU4885 PSU4895	2010	Mussel Mussel Hard clam Hard clam	−	−	0.2	−
				−	−	0.2	−
				−	−	0.1	−
				−	−	0.2	−
	PSU5379	2012	Shellfish	−	−	0.1	−
Clinical isolates							
*tdh* + *trh*− (3)	PSU3872	2008	Clinical	+	+	0.3	+
	PSU3949	2008	Clinical	+	+	0.2	+
	PSU5126	2011	Clinical	+	+	0.1	+
*tdh*− *trh*+ (1)	ATCC17802	1965	Clinical	−	−	0.2	−

* GS-PCR, Group-Specific PCR; MAR, Multiple Antibiotic Resistance.

**Table 2:** Antimicrobial susceptibility patterns of *V. parahaemolyticus* isolates from seafood sources.

Antimicrobial Drugs	No. of Isolates (%)		
	Susceptible	Intermediate	Resistant
Ampicillin	0	0	51 (100)
Gentamycin	12 (23.5)	39 (76.5)	0
Erythromycin	0	29 (56.9)	22 (43.1)
Sulfonamide	49 (96)	1 (2)	1 (2)
Tetracycline	49 (96)	1 (2)	1 (2)
Ciprofloxacin	12 (23.5)	38 (74.5)	1 (2)
Trimethoprim/sulfamethoxazole	36 (70.6)	13 (25.5)	2 (3.9)
Chloramphenicol	51 (100)	0	0
Norfloxacin	34 (66.7)	16 (31.3)	1 (2)

**Table 3:** Characteristics of CRISPR repeat sequences and pattern in CRISPR-positive *V. parahaemolyticus* isolates.

Hemolysin Genotype (*n*)	Type	Direct Repeats (DRs) Sequences *	No. of DRs	No. of Spacers (Sp Type)	CRISPR Locus Pattern (bp) *
Seafood isolates					
*tdh+ trh*− (15)	Typical repeat Terminal repeat	GTGAACTGCCGAATAGGTAGCTGATAAT GTGAACTGCCGCATAGGTAGAGAGAATC	3	2 (1, 4)	*28*-**32***-28*-**31**-*28*
*tdh*− *trh*+ (2)	Typical repeat Terminal repeat	GTGAACTGCCGAATAGGTAGCTGATAAT GTGAACTGCCGCATAGGTAGAGAGGATC	3	2 (2, 4)	*28*-**31**-*28*-**31**-*28*
*tdh+ trh*+ (1)	Typical repeat Terminal repeat	GTGAACTGCCGAATAGGTAGCTGATAAT GTGAACTGCCGCATAGGTAGAGAGGATC	2	1 (3)	*28*-**31**-*28*
*tdh*− *trh*− (1)	Typical repeat Terminal repeat	GTGAACTGCCGAATAGGTAGCTGATAAT GTGAACTGCCGAATAGGTAGAGAGGATC	2	1 (1)	*28*-**32**-*28*
Clinical isolates					
*tdh* + *trh*− (3)	Typical repeat Terminal repeat	GTGAACTGCCGAATAGGTAGCTGATAAT GTGAACTGCCGCATAGGTAGAGAGAATC	3	2 (1, 4)	*28*-**32**-*28*-**31**-*28*

* Underline indicates mutation compared to typical repeat sequence, italic indicates the direct repeats (CDRs) sequence length and bold indicates the spacer length.

**Table 4:** Homology analysis of *V. parahaemolyticus* spacers against foreign genetic elements.

Spacer Type (Length)	Spacer Sequences (5′ to 3′)	Spacer Homology	Query Cover/ Identities (%)	Accession Number/ Location
Sp1 (32)	GAGATACCACAAGCTCAAGCAGATGCTAACAG	*Vibrio alginolyticus* strain K08M3 plasmid pL300	93/96.7	CP017915.1/ 184601-184630
Sp2 (31)	TCATTCTCACGATCTAATTACAGTTGGTCAC	*Vibrio alginolyticus* strain K08M3 plasmid pL300	93/100	CP017915.1/ 155899-155927
Sp3 (31)	TGCAGACAAACAAAGAGCCATCGACGAGTGC	*Vibrio alginolyticus* strain K08M3 plasmid pL300	100/87.1	CP017915.1/ 141985-142015
Sp4 (31)	AGTCGGTCAACTGAGAATACGTTGTTGCCAA	-	-	-

**Table 5:** Homology analysis of the Sp1 to Sp4 spacers against public genomes of *V. parahaemolyticus*.

Spacer Type	Isolates	Hemolysin Genotype *	Sources *	Identities (%)	E-Value	Accession Number
Sp1	*Vibrio parahaemolyticus* strain RIMD 2210633	*tdh*+ *trh*−	Clinical, Japan	100	2 × 10^−8^	BA000032.2
	*Vibrio parahaemolyticus* strain VPD14	*tdh*+ *trh*−	Shrimp, China	100	2 × 10^−8^	CP031782.1
	*Vibrio parahaemolyticus* strain FDAARGOS_191	*tdh*+ *trh*−	Clinical, India	100	2 × 10^−8^	CP020428.2
	*Vibrio parahaemolyticus* strain BB220P	*tdh*+ *trh*−	Environment, India	96.88	1 × 10^−6^	CP003973.1
	*Vibrio parahaemolyticus* strain FORC_071	*tdh*+ *trh*−	Clinical, South Korea	100	1 × 10^−6^	CP023486.1
Sp2	*Vibrio parahaemolyticus* strain Vp17	*tdh*^unk^ *trh*^unk^	Clam, India	100	7 × 10^−8^	MG765521.1
	*Vibrio parahaemolyticus* strain Vp14	*tdh*^unk^ *trh*^unk^	Oyster, India	100	7 × 10^−8^	MG765520.1
	*Vibrio parahaemolyticus* strain Vp8	*tdh*^unk^ *trh*^unk^	Shrimp, India	100	9 × 10^−7^	MG765517.1
Sp3	*Vibrio parahaemolyticus* strain 10329	*tdh*+ *trh*+	Clinical, USA	100	7 × 10^−8^	CP045795.1
	*Vibrio parahaemolyticus* strain MAVP-26	*tdh*+ *trh*+	Clinical, USA	100	7 × 10^−8^	CP023247.1
	*Vibrio parahaemolyticus* strain ST631	*tdh*+ *trh*+	Clinical, USA	100	7 × 10^−8^	CP011885.1
	*Vibrio parahaemolyticus* strain MAVP-QPI	*tdh*+ *trh*+	Clinical, USA	100	7 × 10^−8^	MF066646.1
	*Vibrio parahaemolyticus* strain MAVP-Q	*tdh*+ *trh*+	Clinical, USA	100	7 × 10^−8^	CP022472.1
	*Vibrio parahaemolyticus* strain FDAARGOS_662	*tdh*+ *trh*+	Clinical, USA	100	7 × 10^−8^	CP044070.1
	*Vibrio parahaemolyticus* strain FDAARGOS_51	*tdh*+ *trh*+	Clinical, USA	100	7 × 10^−8^	CP026042.1
	*Vibrio parahaemolyticus* strain 2014V-1125	*tdh*+ *trh*+	Clinical, USA	100	7 × 10^−8^	CP046777.1
	*Vibrio parahaemolyticus* strain 2014V-1066	*tdh*+ *trh*+	Clinical, USA	100	7 × 10^−8^	CP046780.1
	*Vibrio parahaemolyticus* strain 2015AW-0174	*tdh*+ *trh*+	Clinical, USA	100	7 × 10^−8^	CP046753.1
	*Vibrio parahaemolyticus* strain 2010V-1106	*tdh*^unk^ *trh*+	Clinical, USA	100	7 × 10^−8^	CP046827.1
	*Vibrio parahaemolyticus* strain 2013V-1146	*tdh*^unk^ *trh*+	Clinical, USA	100	7 × 10^−8^	CP046809.1
	*Vibrio parahaemolyticus* strain 2013V-1181	*tdh*^unk^ *trh*+	Clinical, USA	100	7 × 10^−8^	CP046784.1
Sp4	*Vibrio parahaemolyticus* strain RIMD 2210633	*tdh*+ *trh*−	Clinical, Japan	100	7 × 10^−8^	BA000032.2
	*Vibrio parahaemolyticus* strain VPD14	*tdh*+ *trh*−	Shrimp, China	100	7 × 10^−8^	CP031782.1
	*Vibrio parahaemolyticus* strain FDAARGOS_191	*tdh*+ *trh*−	Clinical, India	100	7 × 10^−8^	CP020428.2
	*Vibrio parahaemolyticus* strain BB22OP	*tdh*+ *trh*−	Environment, Bangladesh	100	7 × 10^−8^	CP003973.1
	*Vibrio parahaemolyticus* strain FORC_071	*tdh*+ *trh*−	Clinical, South Korea	100	7 × 10^−8^	CP023486.1
	*Vibrio parahaemolyticus* strain Vp14	*tdh*^unk^ *trh*^unk^	Oyster, India	100	7 × 10^−8^	MG765520.1
	*Vibrio parahaemolyticus* strain Vp9	*tdh*^unk^ *trh*^unk^	Oyster, India	100	7 × 10^−8^	MG765518.1
	*Vibrio parahaemolyticus* strain Vp8	*tdh*^unk^ *trh*^unk^	Shrimp, India	100	7 × 10^−8^	MG765517.1

* Hemolysin genotypes and sources were indicated based on the results obtained by database search and/or previous publications. Unk—unknown.

## Data Availability

All relevant data are within the paper and its Supplementary Files.

## Supplementary Materials

The following are available online at https://www.mdpi.com/article/10.3390/microorganisms9061220/s1, Figure S1: The multiple genome alignment by the progressiveMauve between *V. alginolyticus* pL300 megaplasmid (top) and other plasmids from the blast hit results. Homologous genomes are indicated by the connected-with-lines collinear blocks, Table S1: Details regarding the conserved CDSs residing in the locally collinear blocks (LCBs) obtained from ProgressiveMauve analysis.
