# Supplementary material for: Characterization and Analysis of Clustered Regularly Interspaced Short Palindromic Repeats (CRISPRs) in Pandemic and Non-Pandemic Vibrio parahaemolyticus Isolates from Seafood Sources
Source: Microorganisms. 2021 Jun 4;9(6):1220. doi: 10.3390/microorganisms9061220 (PMC8226915; doi:10.3390/microorganisms9061220)
Supplement: Supplementary file 1 [file microorganisms-09-01220-s001.zip › Supplementary Figure S1.pdf]

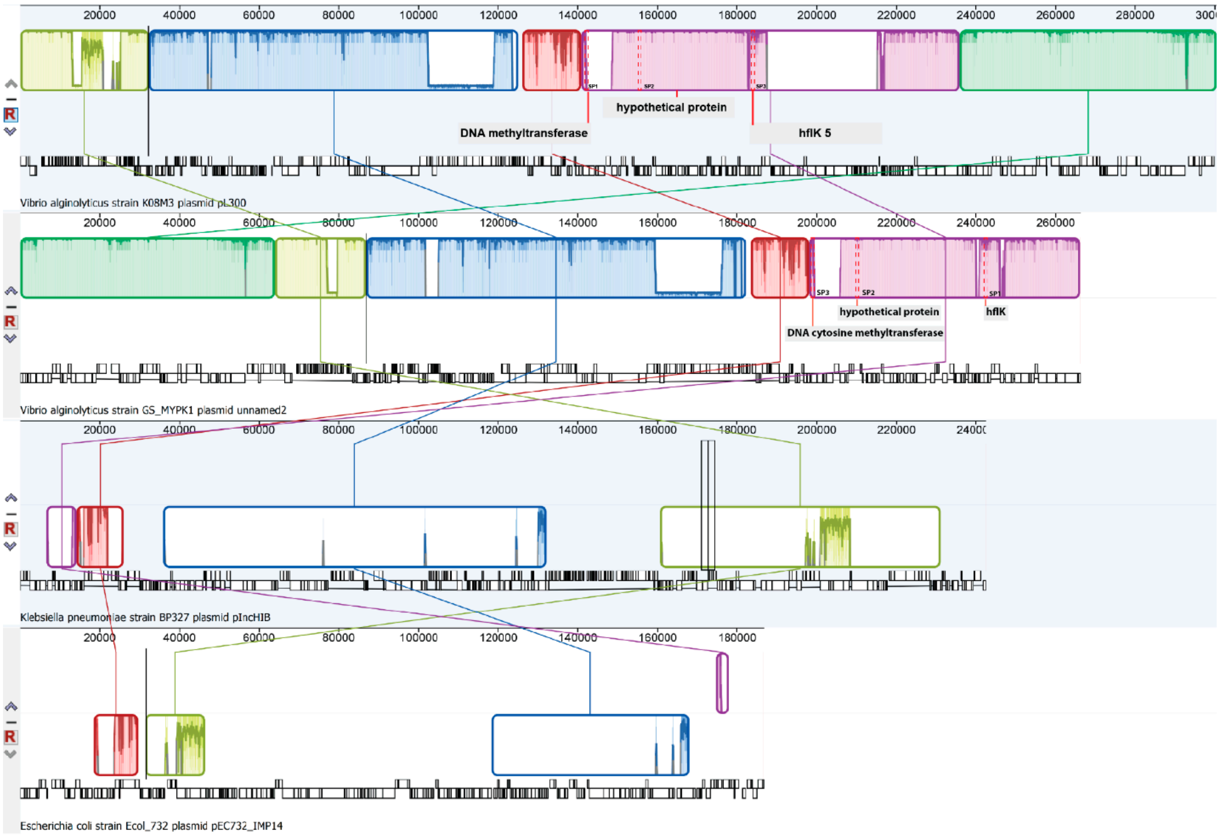

Figure S1. The multiple genome alignment by the progressiveMauve between *V. alginolyticus* pL300 megaplasmid (top) and other plasmids from the blast hit results. Homologous genomes are indicated by the connected-with-lines collinear blocks.
