# Supplementary material for: Characterization and Analysis of Clustered Regularly Interspaced Short Palindromic Repeats (CRISPRs) in Pandemic and Non-Pandemic Vibrio parahaemolyticus Isolates from Seafood Sources
Source: Microorganisms. 2021 Jun 4;9(6):1220. doi: 10.3390/microorganisms9061220 (PMC8226915; doi:10.3390/microorganisms9061220)
Supplement: Supplementary file 1 [file microorganisms-09-01220-s001.zip › Supplementrary Table S1.pdf]

Table S1. Details regarding the conserved CDSs residing in the locally collinear blocks (LCBs) obtained from ProgressiveMauve analysis.

LCB1: Red (29 kbp)	LCB2: Green lime (105 kbp)	LCB3: Green (65 kbp)	LCB4: Blue (241 kbp)	LCB5: Purple (104 kbp)
• type I restriction endonuclease subunit M CDS<br>• DNA/RNA non-specific endonuclease CDS<br>• DNA/RNA non-specific endonuclease CDS<br>• transcriptional regulator CDS<br>• H-NS histone family protein CDS<br>• porin family protein CDS<br>• transcriptional regulator CDS<br>• H-NS histone family protein CDS<br>• ANT(3'') family aminoglycoside nucleotidyltransferase CDS<br>• aadA CDS<br>• aadA gene<br>• DUF1010 domain-containing protein CDS<br>• class D beta-lactamase CDS<br>• dfrA12 CDS<br>• dfrA12 gene<br>• chloramphenicol efflux MFS transporter CDS<br>• GNAT family N-acetyltransferase CDS<br>• rifampin ADP-ribosylating transferase ARR-2 CDS<br>• intI1 CDS<br>• intI1 gene<br>• aminoglycoside nucleotidyltransferase CDS<br>• transposase CDS<br>• ethidium bromide resistance protein CDS<br>• recombinase family protein CDS	• plasmid SOS inhibition protein A CDS<br>• DUF1173 family protein CDS<br>• nuclease CDS<br>• DNA repair protein CDS<br>• DNA-binding protein H-NS-like protein CDS<br>• ISNCY-like element ISKpn21 family transposase CDS<br>• ISL3-like element ISKox3 family transposase CDS<br>• thioredoxin CDS<br>• thiol:disulfide interchange protein CDS<br>• lytic transglycosylase CDS<br>• viral (Super1) RNA helicase CDS<br>• MFS transporter CDS<br>• efflux transporter outer membrane subunit CDS<br>• multidrug efflux RND transporter permease subunit CDS<br>• transcriptional regulator CDS<br>• efflux RND transporter periplasmic adaptor subunit CDS<br>• response regulator transcription factor CDS<br>• HAMP domain-containing protein CDS<br>• diguanylate cyclase CDS<br>• AraC family transcriptional regulator CDS<br>• EamA family transporter CDS<br>• LuxR family transcriptional regulator CDS	• Helix-turn-helix domain protein CDS<br>• DGQHR domain-containing protein CDS<br>• phosphoadenosine phosphosulfate reductase family protein CDS<br>• CobQ/CobB/MinD/ParA nucleotide binding domain protein CDS<br>• ParA family protein CDS<br>• HipA domain-containing protein CDS<br>• uvrD1 CDS<br>• uvrD1 gene<br>• ATP-dependent helicase CDS<br>• StbA plasmid stability protein CDS<br>• ParM/StbA family protein CDS<br>• DNA polymerase III subunit epsilon CDS<br>• 3'-5' exonuclease CDS<br>• DUF3346 domain-containing protein CDS<br>• dnaG 2 CDS<br>• dnaG 2 gene<br>• toprim domain-containing protein CDS<br>• recombination associated protein CDS<br>• rdgC CDS<br>• rdgC gene<br>• DotI/IcmL/TraM family protein CDS	• helix-turn-helix domain-containing protein CDS<br>• type II secretion system protein CDS<br>• P-type DNA transfer ATPase VirB11 CDS<br>• tadA CDS<br>• tadA gene<br>• bfpB CDS<br>• bfpB gene<br>• pilT CDS<br>• pilT gene<br>• tadA CDS<br>• tadA gene<br>• type IV secretory system conjugative DNA transfer family protein CDS<br>• DEAD/DEAH box helicase family protein CDS<br>• DNA topoisomerase III CDS<br>• topA CDS<br>• topA gene<br>• transcriptional regulator CDS<br>• diaminopimelate decarboxylase CDS<br>• transcriptional regulator CDS<br>• chromosome segregation protein ParM CDS<br>• DNA-binding protein HU CDS<br>• nuclease CDS<br>• nitrite reductase CDS<br>• Fe3+-siderophore ABC transporter permease CDS<br>• protein-disulfide isomerase CDS<br>• peptidase S49 CDS<br>• phosphohydrolase CDS<br>• plasmid replication protein CDS	• AAA-like domain protein CDS<br>• Trypsin CDS<br>• trypsin-like serine protease CDS<br>• adenylosuccinate synthase CDS<br>• cyclic-di-GMP phosphodiesterase CDS<br>• EAL domain-containing protein CDS<br>• EAL domain-containing protein CDS<br>• N-6 DNA methylase CDS<br>• AAA-like domain protein CDS<br>• Trypsin CDS<br>• EAL domain-containing protein CDS<br>• prmC 2 CDS<br>• prmC 2 gene<br>• Y-family DNA polymerase CDS<br>• XRE family transcriptional regulator CDS<br>• DNA polymerase V CDS<br>• hflK 5 CDS<br>• hflK 5 gene<br>• hflK CDS<br>• hflK gene<br>• hflC 3 CDS<br>• hflC 3 gene<br>• hflC CDS<br>• hflC gene<br>• restriction endonuclease CDS<br>• DNA methyltransferase CDS<br>• DNA cytosine methyltransferase CDS<br>• HNH endonuclease CDS<br>• GIY-YIG nuclease family protein CDS

• integrase CDS<br>• transcriptional regulator CDS<br>• transposase CDS<br>• Tn3 family transposase CDS<br>• IS110-like element IS4321 family transposase CDS<br>• replication initiation protein CDS<br>• RNA polymerase-binding transcription factor CDS<br>• transposase CDS<br>• recombination regulator RecX CDS<br>• RecX family transcriptional regulator CDS<br>• transposase CDS	• Phage integrase family protein CDS<br>• AsnC family protein CDS<br>• tyrosine--tRNA ligase CDS<br>• AAA family ATPase CDS<br>• DNA primase CDS<br>• NgrC CDS<br>• integrase CDS<br>• pyruvate dehydrogenase CDS<br>• 5'-deoxynucleotidase CDS<br>• site-specific integrase CDS<br>• conjugal transfer protein TraG CDS<br>• conjugal transfer protein CDS<br>• thioredoxin family protein CDS<br>• transposase CDS<br>• conjugal transfer protein TraI CDS<br>• transposase CDS<br>• conjugal transfer protein TraD CDS<br>• Archaeal ATPase CDS<br>• AAA family ATPase CDS<br>• DUF4400 domain-containing protein CDS<br>• S49 family peptidase CDS<br>• NERD domain-containing protein CDS<br>• integrase CDS<br>• Initiator Replication protein CDS<br>• integrase CDS		• plasmid stability protein StbA CDS<br>• DNA-binding protein CDS<br>• regulator CDS<br>• transcriptional regulator CDS<br>• transglycosylase CDS<br>• nucleotide excision repair protein CDS<br>• permease CDS<br>• conjugal transfer protein TraG CDS<br>• conjugal transfer protein TraH CDS<br>• conjugal transfer protein TraF CDS<br>• DNA helicase CDS<br>• KfrA protein CDS<br>• DNA replication terminus site-binding protein CDS<br>• DNA polymerase III subunit epsilon CDS<br>• integrase CDS<br>• topoisomerase CDS<br>• VWA domain-containing protein CDS<br>• cell surface protein CDS<br>• DUF3150 domain-containing protein CDS<br>• ATP-binding protein CDS<br>• ATP-dependent helicase CDS<br>• type II toxin-antitoxin system Phd/YefM family antitoxin CDS<br>• type II toxin-antitoxin system RelE/ParE family toxin CDS<br>• IS200/IS605 family transposase CDS<br>• transposase CDS<br>• transposase CDS<br>• tnpA CDS<br>• tnpA gene<br>• transposase CDS<br>• conjugal transfer protein TraG CDS	• Phage integrase family protein CDS<br>• DUF2971 domain-containing protein CDS<br>• DUF4365 domain-containing protein CDS<br>• ProQ/FINO family protein CDS<br>• IS4-like element ISEc29 family transposase CDS<br>• ArmA family 16S rRNA (guanine(1405)-N(7))-methyltransferase CDS<br>• IS5 family transposase CDS<br>• IS91 family transposase CDS<br>• transposase CDS<br>• xerC CDS<br>• xerC gene<br>• site-specific integrase CDS<br>• ATP:cob(I)alamin adenosyltransferase CDS<br>• EAL domain-containing protein CDS<br>• bifunctional diguanylate cyclase/phosphodiesterase CDS<br>• Phage integrase family protein CDS<br>• tyrosine-type recombinase/integrase CDS<br>• sul1 CDS<br>• sul1 gene<br>• sulfonamide-resistant dihydropteroate synthase Sul1 CDS<br>• DNA methyltransferase CDS<br>• DNA cytosine methyltransferase CDS<br>• quaternary ammonium compound efflux SMR transporter QacE delta 1 CDS<br>• ethidium bromide resistance protein CDS<br>• type I restriction endonuclease subunit M CDS

			• conjugal transfer protein TraN CDS<br>• plasmid transfer protein CDS<br>• conjugal transfer protein CDS<br>• lepB CDS<br>• lepB gene<br>• TraM recognition domain-containing protein CDS<br>• plasmid transfer protein CDS<br>• DotA/TraY family protein CDS<br>• HtdA CDS<br>• TrhO CDS<br>• trhZ CDS<br>• DNA primase CDS<br>• AAA family ATPase CDS<br>• traV CDS<br>• traV gene<br>• DsbC family protein CDS<br>• conjugal transfer protein TraB CDS<br>• DNA (cytosine-5-)-methyltransferase CDS<br>• DNA cytosine methyltransferase CDS<br>• traK family protein CDS<br>• conjugal transfer protein TraE CDS<br>• plasmid transfer protein CDS<br>• pili assembly chaperone CDS<br>• IS5-like element ISKpn26 family transposase CDS<br>• DNA-binding protein CDS<br>• DUF4165 domain-containing protein CDS<br>• ParA family protein CDS<br>• peptide transporter CDS<br>• DUF4165 domain-containing protein CDS<br>• IS3 family transposase CDS	

			• DUF4165 domain-containing protein CDS<br>• DUF4165 domain-containing protein CDS<br>• NYN domain-containing protein CDS<br>• HNH endonuclease CDS<br>• DNA-binding protein CDS<br>• dnaC CDS<br>• dnaC gene<br>• DNA cytosine methyltransferase CDS<br>• restriction endonuclease CDS<br>• DUF3846 domain-containing protein CDS<br>• conjugal transfer protein TraF CDS<br>• Eac protein CDS<br>• 2Fe-2S ferredoxin-like protein CDS<br>• plasmid maintenance system killer family protein CDS<br>• IS6-like element IS26 family transposase CDS<br>• IS6-like element IS26 family transposase CDS<br>• Tn3 family transposase CDS<br>• cupin fold metalloprotein, WbuC family CDS<br>• type II toxin-antitoxin system PemK/MazF family toxin CDS<br>• AbrB/MazE/SpoVT family DNA-binding domain-containing protein CDS<br>• class A extended-spectrum beta-lactamase CTX-M-15 CDS<br>• site-specific integrase CDS<br>• IS1380-like element ISEc9 family transposase CDS	

			• DUF4158 domain-containing protein CDS<br>• recombinase family protein CDS<br>• DUF4942 domain-containing protein CDS<br>• type IV secretion protein Rhs CDS<br>• class A broad-spectrum beta-lactamase TEM-1 CDS<br>• IS91 family transposase CDS<br>• aminoglycoside O-phosphotransferase APH(6)-Id CDS<br>• aph(3'') CDS<br>• aph(3'') gene<br>• relE CDS<br>• relE gene<br>• type II toxin-antitoxin system RelE/ParE family toxin CDS<br>• yafN CDS<br>• yafN gene<br>• type II toxin-antitoxin system Phd/YefM family antitoxin CDS<br>• sul2 CDS<br>• sul2 gene<br>• transcriptional regulator CDS<br>• DUF4102 domain-containing protein CDS<br>• IS110-like element IS5075 family transposase CDS<br>• transposase CDS<br>• Tn3 family transposase CDS<br>• AraC family transcriptional regulator CDS<br>• IS6-like element IS26 family transposase CDS<br>• transposase CDS	
